# Changes in the mechanical properties of human mesenchymal stem cells during differentiation

**DOI:** 10.1098/rsos.220607

**Published:** 2023-01-04

**Authors:** Fei He, Chendong Yang, Haoye Liu, Jizeng Wang

**Affiliations:** Key Laboratory of Mechanics on Disaster and Environment in Western China, Ministry of Education, College of Civil Engineering and Mechanics, Lanzhou University, Lanzhou, Gansu 730000, People's Republic of China

**Keywords:** cell differentiation, power-law rheology model, viscoelasticity, human mesenchymal stem cells

## Abstract

A thorough understanding of the changes in mechanical property behind intracellular biophysical and biochemical processes during differentiation of human mesenchymal stem cells (hMSCs) is helpful to direct and enhance the commitment of cells to a particular lineage. In this study, displacement creep of the mesenchymal cell lineages (osteogenic, chondrogenic and adipogenic hMSCs) were determined by using atomic force microscopy, which was then used to determine their mechanical properties. We found that at any stages of differentiation, the mesenchymal cell lineages are linear viscoelastic materials and well matched with a simple power-law creep compliance. In addition, the viscoelasticity of mesenchymal cell lineages showed different trends during differentiation. The adipogenic hMSCs showed continuous softening at all stages. The osteogenic and chondrogenic hMSCs only continuously soften and become more fluid-like in the early stage of differentiation, and get stiffened and less fluid-like in the later stage. These findings will help more accurately imitate cellular biomechanics in the microenvironment, and provided an important reference in the biophysics biomimetic design of stem cell differentiation.

## 1. Introduction

Human mesenchymal stem cells (hMSCs) can be obtained from the bone marrow, adipose tissue and umbilical cords. These cells can be expanded *in vitro* and differentiate into multiple lineages, including chondroblasts, osteoblasts and adipocytes [1,2]. The application of hMSCs not only eliminates ethical issues but also provides a new method for the regeneration and repair of bone, cartilage, muscle and nerve tissue [3]. Biomechanical studies on the differentiation of hMSCs are common. For example, Darling *et al*. found that different lineages of differentiated hMSCs exhibit distinct mechanical properties; that is, osteoblasts are stiffer than chondrocytes, and both are stiffer than adipocytes [4]. Yu *et al*. claimed that the hMSCs gradually stiffen with osteogenic differentiation, but stiffen on days 0–7 and then soften on days 7–21 during adipogenic differentiation [5]. However, Titushkin *et al*. also indicated that hMSCs gradually soften during osteogenic differentiation [6]. Thus, the unique biomechanical properties of hMSCs during differentiation have not been conclusive. Matching the mechanical properties of hMSCs to those of fully differentiated cells is crucial for the development of functional load-bearing connective tissue. Sen *et al.* used cytochalasin D (an inhibitor of the microfilament skeleton structure) to depolymerize filamentous actin of hMSC structure into globular-actin (G-actin). And the intranuclear G-actin forces hMSCs into osteogenic lineage [7]. Kuo *et al*. indicated that oscillatory shear stress could induce directional reorganization of F-actin to mediate the fate choice of hMSCs through the regulation of β-catenin [8]. Thus, cellular microfilament skeleton structure plays a significant role in regulating the hMSC lineage. As an important feature of the microfilament skeleton, its biomechanical properties should be quantified accurately. Meanwhile, tissue stiffness of different differentiation origins considerably varies. Nevertheless, cross-sectional studies on the biomechanical properties of hMSCs in the different differentiated lineages have been ignored, which should have great application potential in the identification of cells.

Given that cells are complex living materials that exhibit both the solid-like property of elasticity and the fluid-like behaviour of viscosity, the cytoskeleton viscoelasticity has been increasingly measured to characterize cells, especially tumor cells, chondrocytes and other cells with strong secretion activity [9–11]. Several techniques, such as atomic force microscopy (AFM), quasi-three-dimensional cell microscopy, magnetic resonance elastography and magnetic or optical tweezers, have been successfully applied to study the viscoelastic properties of cells [12–14]. Among all methods, indentation techniques based on the AFM are most popularly adopted. AFM systems facilitate the high-resolution imaging of samples and quantify loading while providing a wide range for force measurement and a rapid feedback control on force and deformation. The creep experiment is one of the most commonly used methods to measure the viscoelasticity of cells through AFM [15,16]. Traditional viscoelastic models consist of a finite number of springs and dashpots to characterize both fluidity and elasticity of cells, where the spring is the ideal linear elastic unit with elastic force being proportional to its deformation, and the dashpot is the ideal linear viscous unit with viscous force being proportional to the deformation rate [17,18]. The cytoskeletons of cells are usually complex polymer networks consisting of polymers with randomly distributed lengths, orientations and bending rigidities. These characteristics make the typical dashpot-type creep timescales statistically distributed and infinite in number. Interestingly, regardless of the cell types or states, cells exhibit a universal power-law rheological behaviour. Contrary to the classical spring-dashpot models that only have a finite number of creep timescales, the power-law rheology (PLR) model can be regarded as a combination of an infinite number of classical springs and dashpots. Thus, the viscoelastic property characterized by this model matches very well that expressed by cells. The applicability of the PLR model in characterizing the viscoelastic property of cells has been successfully demonstrated in existing studies [19,20].

In this study, the creep behaviour of hMSCs during the whole process of osteogenic, adipogenic and chondrogenic differentiation was measured by using AFM, the quantitative data were obtained through ramp-hold processing in combination with a PLR model. Moreover, the viscoelastic properties of long-term cultured undifferentiated hMSCs were further investigated to compare with those of differentiated cells. This work explored the exact conditions to simulate the mechanical microenvironment in the musculoskeletal tissues and has great value in the guidance of the bionic design for tissue engineering.

## 2. Method and materials

### 2.1. Cell culture and differentiation

The hMSCs (Cyagen Biosciences, Guangzhou, China) were cultured in α-MEM (Hyclone, Logan, USA) supplemented with 10% fetal bovine serum (FBS; Gibco, USA), 1% penicillin-streptomycin (Hyclone, Logan, USA) and 1% glutamine (Hyclone, Logan, USA) at 37°C in a humidified atmosphere containing 5% CO_2_. After the monolayer of adherent cells had reached confluence, the cells were trypsinized (0.25% trypsin/EDTA; Hyclone, Logan, USA) and subcultured at the density of 5000 cells cm^−2^. Then, the medium was changed every 48 h with the same composition. The third to eighth passages of the hMSCs were used for the experiment.

The hMSCs were seeded and subjected to induction on sterilized 35 mm cell culture dishes (Beaverbio, Suzhou, China). After 80–90% confluence was attained, the hMSCs osteodifferentiation of the hMSCs was induced in the osteogenic medium (OM) supplemented with 10% FBS, 0.1 µM dexamethasone (Hyclone, Logan, USA), 10 mM β-glycerophosphate (Aladdin, Shanghai, China) and 50 µM ascorbic acid (Aladdin, Shanghai, China) in α-MEM (Hyclone, Logan, USA). Adipogenic induction was induced in the adipogenic medium (AM) supplemented with 10% FBS, 0.5 mM isobutylmethylxanthine (Aladdin, Shanghai, China), 10 µg ml^−1^ insulin (Sigma-Aldrich, St. Louis, USA) and 10^−6^ M dexamethasone in low-glucose DMEM (Hyclone, Logan, USA). Chondrogenic induction was induced in the chondrogenic medium (CM) supplemented with 1% ITS (Sigma-Aldrich, St. Louis, USA), 10^−7^ M dexamethasone, 1 mM sodium pyruvate (Aladdin, Shanghai, China), 120 µM ascorbic acid (Hyclone, Logan, USA), 100 µM non-essential amino acids and 10 ng ml^−1^ transforming growth factor-beta 3 (TGF-β3, Cell inspire Bio, Shenzhen, China) in high-glucose DMEM (Hyclone, Logan, USA). To verify the validity of the differentiation experiments, alizarin red staining, alcian blue staining and Nile red fluorescent staining were used to verify the validity of the osteogenic, chondrogenic and adipogenic differentiation, respectively. Induced hMSCs were washed with DPBS and fixed with 4% (v/v) formaldehyde at room temperature for 20 min. Specifically, as to alizarin red staining, 40 mM Alizarin Red-S was added into cultures for staining (pH = 7.4, AS, Solarbio, Beijing, China), after 20 min of incubating and three times of washing, the excess water was removed and the cultures were observed under phase contrast microscopy (NA 0.75, 20×; Olympus, Tokyo, Japan). As to alcian blue staining, the chondrogenic hMSCs were washed with DPBS three times before the addition of Alcian Blue Cartilage Stain Solution, pH = 1.0 (Solarbio, Beijing, China), the cultures were scanned under a phase contrast microscope (NA 0.75, 20×) after 30 min of incubating and three times of washing. As to Nile red fluorescent staining, the adipogenic induced cells were washed three times and stained with 1 µM Nile red (Solarbio, Beijing, China) with Hoechst 33342 (Solarbio, Beijing, China) for 5 min in room temperature [21]. After three times of washing, a fluorescence microscope (NA 0.9, 40×, Olympus, Tokyo, Japan) was used for scanning and photography.

### 2.2. Characterization of cell viscoelasticity

Creep assays were conducted on the hMSCs during particular lineage differentiation using a Nanowizard III BioScience AFM (JPK, Berlin, Germany) on days 0, 7, 14, 21 and 28 to characterize the viscoelasticity. Meanwhile, long-term culture (28 days) without differentiation was set as the control. All AFM experiments were carried out at room temperature within 1 h per dish to ensure the bioactivity of the hMSCs. The probe was a polystyrene bead with a diameter of 4.5 µm attached to a triangular silicon nitride cantilever with a nominal spring constant of 0.01 N m^−1^ (Novascan, Boone, USA). The cantilever was calibrated in the dishes by using the thermal noise method before measurement [22]. Undifferentiated hMSCs on day 0 were used to explore the effect of loading rate and maximal force on the results, the ramp rate of the probe was set to 1–10 µm s^−1^ for indentation until the cantilever deflection reached 0.5–2 nN. The deflection was held constant for 10 s. Each cell was indented at its nuclear region only once to reduce heterogeneity. For each experimental group, more than 40 cells were measured to ensure the stability of the average results.

**Figure 1.**
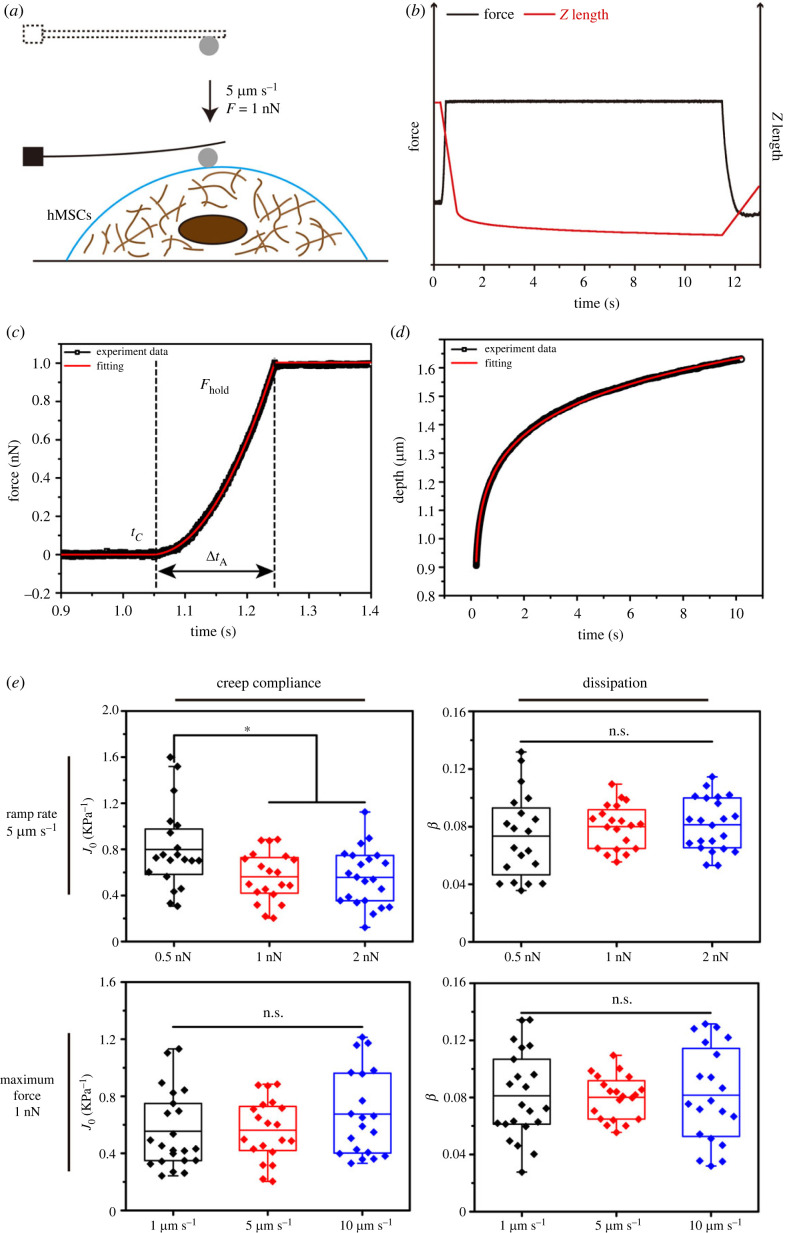
Schematic of the creep experiments on the hMSCs. (*a*) Schematic of the indentation experiment. (*b*) Indentation force and Z-position of the cantilever base in the creep experiments. (*c,d*) Loading history fitted by equation (2.4). (*e*) Least-square fitting parameters of undifferentiated hMSCs with series of loading velocities and forces. * (*p* < 0.05), n.s. (*p* > 0.05).

The schematic of the indentation experiment is shown in figure 1*a*. In the AFM indentation experiments, the cells were assumed to be incompressible linear viscoelastic materials, so that their constitutive relation can be expressed as follows [23]:

2.1 $$\varepsilon_{ij}(t)=\int_{0}^{t}J(t-\tau )\frac{\partial \sigma_{ij}(\tau )}{\partial \tau }d\tau ,$$

where J(t) is the creep compliance, $\varepsilon_{ij}$ and $\sigma_{ij}$ are the strain and stress components associated with the *i* and *j* directions. The creep experiment to hMSCs by using AFM with a spherical probe could be viewed as a rigid spherical indenter to vertically contact with a viscoelastic half-space. On the basis of the contact theory of viscoelastic materials [24], the relation between the loading force F(t) of the spherical probe and the corresponding indentation depth in the cell, δ(t), can be expressed as follows [24]:

2.2 $$\delta^{3/2}(t)=\frac{3}{8\sqrt{R}}\int_{0}^{t}J(t-\tau )\frac{\partial F(\tau )}{\partial \tau }d\tau .$$

For the creep compliance J(t), the PLR model was adopted as follows [25]:

2.3 $$J(t)=J_{0}{\left(\frac{t}{\tau_{0}}\right)}^{\beta },$$

in which the prefactor $J_{0}=1/E_{0}$ can be viewed as the inverse of the Young's modulus, $E_{0}$, and characterizes the stiffness of the material at time $t=\tau_{0}$. The power-law exponent β∈( 0,1)  characterizes the degree of dissipation or ‘fluidity’ of the viscoelastic material [25,26].

A so-called ramp-hold method was adopted for the AFM indentation experiments [27], which consisted of two phases, the ramp and the hold. For simplicity, a constant ramp rate, 5 µm s^−1^, of the AFM probe was used in the ramp phase. Under such a slow rate of ramp, the commonly used step-hold method [4] of instantaneous loading may lead to erroneous estimation on the viscoelastic properties.

On the basis of the PLR model, the calibration formula for each measured force curve can be expressed as follows [27]:

2.4 $$F(t)=F_{\mathrm{hold}}\{\begin{array}{cc} 0 & t<t_{c}\\ {(\frac{t-t_{c}}{\Delta t_{A}})}^{a} & t_{c}\leq t<t_{c}+\Delta t_{A}\\ 1 & t_{c}+\Delta t_{A}\leq t \end{array}$$

where *t_c_*, $\Delta t_{A}$, *a* and $F_{\mathrm{hold}}$ are the fitting parameters for the history of loading force (figure 1*c*).

Once the contact point, $\left(t_{c},Z_{c}\right)$, on each force curve is determined, the indentation depth δ during the hold phase can be obtained as follows:

2.5 $$\delta =Z-Z_{c}-\frac{F_{\mathrm{hold}}}{k}$$

where *Z* is the overall displacement of the probe (figure 1*b*), and *k* is the spring constant of the probe cantilever. By setting $\tau_{0}=1s$ and inserting equations (2.3) and (2.4) into equation (2.2), the formula of data fitting for the hold phase can be derived as follows [27]:

2.6 $$\delta^{3/2}(t)=\begin{array}{cc} \frac{3aF_{\mathrm{hold}}}{8\sqrt{R}{\left(\Delta t_{A}\right)}^{a}}J_{0}{\left(t-t_{c}\right)}^{a+\beta }B\left(\frac{\Delta t_{A}}{t-t_{c}};\;a,1+\beta \right) & t\geq t_{c}+\Delta t_{A} \end{array},$$

where $B(\Delta t_{A}/(t-t_{c});a,1+\beta )$ is the incomplete Beta function. The remaining free parameters *J*_0_ and *β* can be obtained by fitting equation (2.6) in terms of the least square method (figure 1*d*). Briefly, in the PLR model, *J*_0_ is the creep modulus of the cells and characterizes the softness of materials, and *β* characterizes the degree of dissipation or fluidity of the viscoelastic material. The cells' behaviour is more fluid-like when *β* approaches 1, and more solid-like when *β* is close to 0.

### 2.3. Statistical analysis

Statistical analysis was performed with SPSS 17. All least-square fitting parameters for the power-law rheology model were presented as the mean ± standard deviation. Statistical significance was tested by one-way ANOVA and differences were considered at *p* < 0.05.

## 3. Results and discussion

Although significant progress has been made in the characterization of cell biomechanics, given the synthesis of fluid-like proteins and the solid-like structures of cells, viscoelasticity should be a more comprehensive parameter for evaluating the cellular biomechanical behaviour during differentiation. Cells exhibit a universal power-law rheological behaviour, and the PLR was developed to describe the viscoelastic behaviour of linear viscoelastic materials. This property could account for the essential mechanical responses of the cell membrane and cytoskeleton [9,25]. A review of literature showed no data was found on the creep compliance and the fluidity of the differentiated hMSCs based on the PLR rheology model. Accurate quantitative investigation of viscoelastic changes during the differentiation of hMSCs could provide a better understanding of the intracellular biophysical process during the differentiation of hMSCs, and hold potential for the identification of hMSCs.

Before characterizing the viscoelastic properties of hMSCs through the technique of AFM indentation, some pivotal parameters were determined by measuring undifferentiated hMSCs in several loading rates and maximal forces. Least-square fitting parameters of undifferentiated hMSCs under the condition of 0.5–2 nN maximal forces and 1–10 µm s^−1^ loading rates were shown in figure 1*e*. When the loading force was 1 nN, the least-square fitting parameters at 10 µm s^−1^ are slightly higher than those of the other groups, which might be caused by the viscous effects of solution and the cell under the intervention of excessive loading speed [6]. However, with a loading rate of 5 µm s^−1^, the 1 nN and 2 nN groups showed almost the same results; in comparison to other groups, the creep compliance of 0.5 nN group was significantly different (*p* < 0.05). The reason might be that the loading force of 0.5 nN was too weak to effectively deform cytoskeletons of the cells. Thus, in subsequent research, the ramp rate of the probe was set to 5 µm s^−1^ for indentation until the cantilever deflection reached 1 nN.

**Figure 2.**
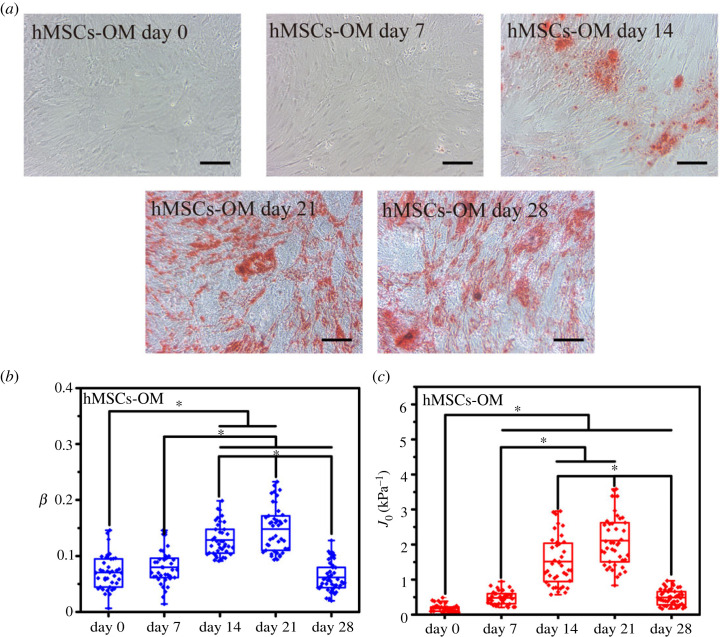
Osteodifferentiation of the hMSCs from days 0 to 28 under the induction of an osteogenic medium. (*a*) Alizarin red staining of hMSCs from days 0 to 28 under the induction of an osteogenic medium. (*b*) Fluidity and (*c*) creep compliance of the osteodifferentiated hMSCs from days 0 to 28. Bar = 100 µm. * (*p* < 0.05).

**Figure 3.**
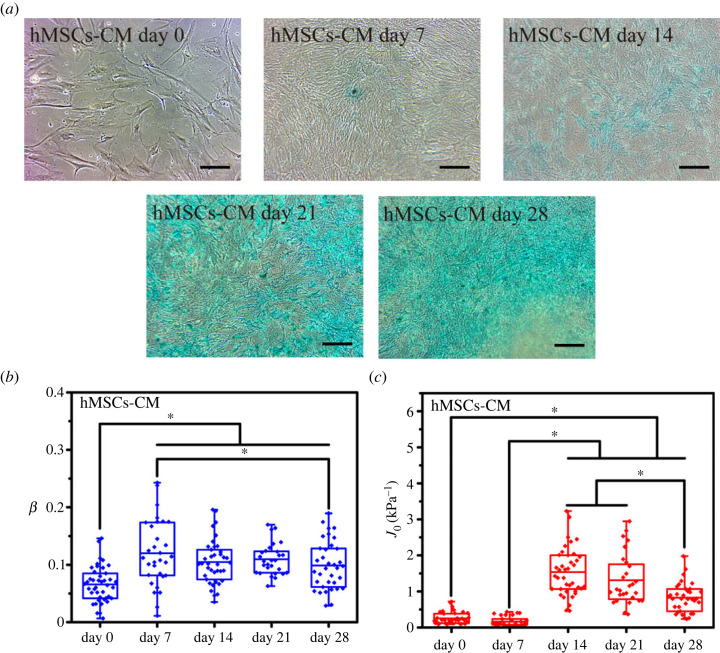
Chondrogenic differentiation of the hMSCs from days 0 to 28 under the induction of a chondrogenic medium. (*a*) Alcian blue staining of hMSCs from days 0 to 28 under the induction of a chondrogenic medium. (*b*) Fluidity degree and (*c*) creep compliance of chondrogenic differentiated hMSCs from days 0 to 28. Bar = 100 µm. * (*p* < 0.05).

**Figure 4.**
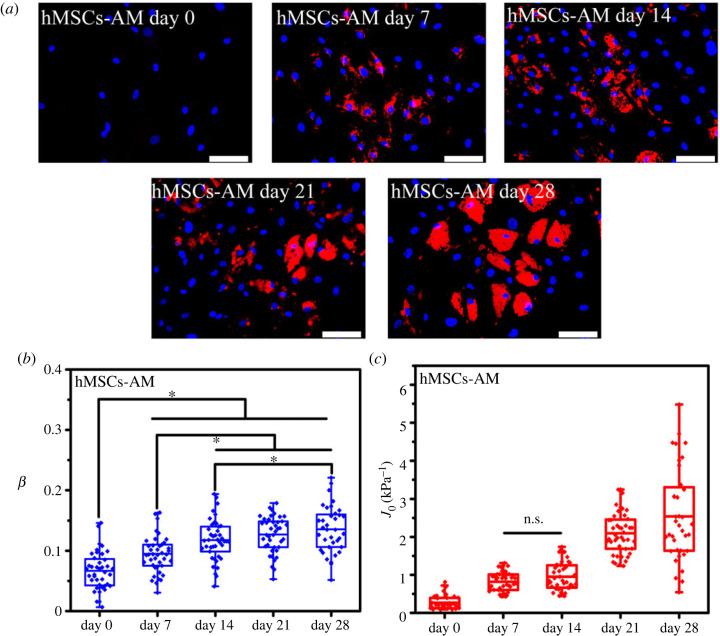
Adipogenic differentiation of the hMSCs from days 0 to 28 under the induction of an adipogenic medium. (*a*) Nile red fluorescent staining of hMSCs from days 0 to 28 under the induction of adipogenic medium. (*b*) Fluidity degree and (*c*) creep compliance of the adipogenic differentiated hMSCs from days 0 to 28. Bar = 50 µm. * (*p* < 0.05), n.s. (*p* > 0.05).

**Figure 5.**
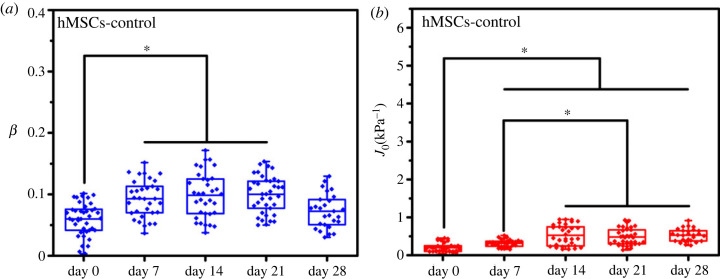
Comparison of fluidity degree and creep compliance of differentiated hMSCs on (*a*) day 7, (*b*) day 14, (*c*) day 21 and (*d*) day 28. * (*p* < 0.05), n.s. (*p* > 0.05).

To characterize the viscoelastic properties of differentiated hMSCs, a differentiation medium was used to induce hMSCs, and the creep behaviour of the differentiated hMSCs was monitored on days 0, 7, 14, 21 and 28 to study the viscoelastic evolution trend of these cells. Positive alizarin red staining, alcian blue staining and Nile red fluorescent staining were performed, and the results are shown in figures 2–4*a*. With the extension of culture time, the stained areas were expanded as previously reported [2,28–30]. The expected stained results indicated the validity of a particular lineage. The least-squares fit parameters for the PLR model are listed in tables 1–3. During the osteodifferentiation of the hMSCs, the fluidity (*β*) did not change significantly within 0–7 days, but gradually increased from day 7 to 21, and then decreased to a relatively low level after continuous incubation for 28 days (*p* < 0.05). The change of creep compliance (*J*_0_) also showed a similar trend, increased before day 21, and reversed from day 21 to 28 significantly (*p* < 0.05, figure 2*b,c*). Past work has indicated that actin filaments are considered to represent the cytoskeletal system that undergoes extensive remodelling during differentiation, and is a major component of the cytoskeleton responsible for cellular stiffness [31]. Therefore, it is comprehensible for creep compliance of hMSCs increased during the early stage of cellular actin remodelling. Lenormand *et al.* confirmed that the fluidity of human airway smooth muscle cells could be weakened by activating their contractile apparatus through histamine, and strengthened by specifically blocking their actin by the cytochalasin D, indicating that cell rheology could arise from cytoskeletal elements agitated and re-arranged through mutual weak interactions within their matrix [32]. In addition, cell differentiation is usually associated with cytoskeleton rearrangement, especially involving the reduction of actin stress fibres or the increase of G-actin/F-actin ratio [6,33,34]. Meanwhile, the expression of osteogenic-related proteins, such as type I collagen, bone sialoprotein, osteonectin and osteocalcin, would cause the formation of vesicles in the cytoplasm. Relevant parts of the cortical actin network would be partially depolymerized when contacting with these vesicles, which is considered an important prerequisite for protein transportation [35,36]. Therefore, the depolymerization of actin and its subsequent cytoskeleton rearrangement might be part of the reason for the change of cell fluidity during osteodifferentiation. However, from day 21 to day 28, *J*_0_ and *β* of the hMSCs decreased rapidly (*p* < 0.05), which may be caused by the condensation and mineral deposition of differentiated hMSCs, resulting in the decrease in stiffness and fluidity (*p* < 0.05) [37]. Interestingly, fluidity of the chondrogenic hMSCs only increased before day 7 (*p* < 0.05), and no specific trend was evident for the other stages (figure 3*b,c*). Unlike fluidity, creep compliance of chondrogenic differentiated hMSCs increased first and then decreased, and the hardening stage of the chondrogenic hMSCs appeared to be earlier than that of osteodifferentiation. The osteogenic and chondrogenic hMSCs had almost the same *J*_0_ on day 14, and the maximum fluidity of chondrogenic hMSCs was reached on day 7 (figure 5*a,b*). These results were not unexpected, because endochondral osteogenesis is the primary mode of bone formation *in vivo*, and cartilage proteoglycans are secreted earlier compared to the formation of the mineralized matrix [38]. However, *J*_0_ and *β* had been increasing almost throughout the whole stage with the induction of adipogenic differentiation accompanied by a further increase in the number and volume of Nile red-stained lipid droplets (figure 4*a–c*), and *β* and *J*_0_ of adipogenic hMSCs were significantly higher than other lineages on day 28 (figure 5*d*). It was widely reported that the actin filaments transform into the discontinuous network-like structure at the cell periphery and around the oil droplets during adipogenic differentiated hMSCs [31]. The migration of actin filaments at the cell periphery and the formation of the internal lipid droplets may be the reason for the continuous increase of both *J*_0_ and *β*. Osteogenic hMSCs appeared to be softer than adipogenic ones on days 14. This trend was quickly reversed on day 28 (figure 5*b–d*). These results indicated the softening and enhanced fluidity of the hMSCs during the early stages of differentiation, while the mechanical properties of the later stages was dependent on the differentiation lineage. Although a large number of studies have been carried out on the biomechanics of hMSCs differentiation, specifically, Titushkin *et al*. and Chen *et al*. claimed that osteodifferentiation led to the gradual reduction in cell elasticity, and the fluorescent staining results indicated that more and more stress fibers are replaced with a thinner actin network as osteogenic differentiation progressed [6,39]. Yu *et al*. substantiated that osteogenic differentiation led to gradual stiffenning of hMSCs [5]. However, our results revealed that viscoelastic changes in the cytoskeleton highly depended on particular lineages. Given the special mineralization mechanism, during the successful osteodifferentiation of the hMSCs, *β* and *J*_0_ increased on days 0–21 and decreased on day 28. Adipogenic differentiation was a process during which liquid-phase lipid droplets assembled and synthesized in cells without mineralization, as evidenced by the absence of a reversal trend in the *J*_0_ and *β*. We note that different techniques of measurements on mechanical properties of cells will involve different cellular states and lead to different quantitative results. For instance, in the works by Titushkin *et al.* and Chen *et al.* the mechanical parameters of attached stem cells were quantified by AFM indentation experiments, whereas the quantitative results of Yu *et al*. were based on micropipette aspiration for the hMSCs under suspended state. Meanwhile, these studies were evaluated at different stages of differentiation, making the relevant results more difficult to compare [5,6,39].

**Table 1.** Least-square fitting parameters for the power-law rheology model of osteodifferentiated hMSCs.

		*J*_0_ (kPa^−1^)	*β*
	day 0	0.203 ± 0.122	0.607 ± 0.026
	day 7	0.478 ± 0.190	0.080 ± 0.031
hMSCs-OM	day 14	1.514 ± 0.694	0.129 ± 0.029
	day 21	2.110 ± 0.717	0.148 ± 0.040
	day 28	0.490 ± 0.227	0.062 ± 0.026

**Table 2.** Least-square fitting parameters for the power-law rheology model of chondrogenic hMSCs.

			*J*_0_ (kPa^−1^)	*β*
		day 0	0.203 ± 0.122	0.607 ± 0.026
		day 7	0.163 ± 0.133	0.120 ± 0.057
hMSCs-CM		day 14	1.538 ± 0.669	0.102 ± 0.037
		day 21	1.314 ± 0.706	0.110 ± 0.027
		day 28	0.826 ± 0.402	0.099 ± 0.042

**Table 3.** Least-square fitting parameters for the power-law rheology model of adipogenic hMSCs.

			*J*_0_ (kPa^−1^)	*β*
		day 0	0.203 ± 0.122	0.607 ± 0.026
		day 7	0.791 ± 0.234	0.096 ± 0.032
hMSCs-AM		day 14	0.927 ± 0.356	0.118 ± 0.033
		day 21	2.089 ± 0.550	0.127 ± 0.030
		day 28	2.542 ± 1.211	0.136 ± 0.038

Many studies have shown that cellular biomechanical properties play an important role in the proliferation, migration, adhesion and differentiation of cells [40,41]. In the past decades, various hypotheses have been proposed to emphasize the relationship between the mechanical properties of the cytoskeleton and cellular differentiation. For example, Titushkin *et al.* demonstrated that the elastic modulus decreased during the osteodifferentiation of hMSCs, and the membrane-cytoskeleton adhesion in osteoblasts is stronger than that in undifferentiated hMSCs. Meanwhile, they claimed that reducing cell stiffness by knocking down Ezrin, Radixin and Moesin families of proteins (ERM) would impair biochemically directed osteodifferentiation [33]. Bergert *et al.* also claimed that apparent membrane tension decreased during the early embryonic stem cell differentiation [42]. Meanwhile, numerous studies have found that adipogenic differentiation decreases the elasticity of hMSCs [5]. Maeda *et al.* claimed that chondrogenic differentiation caused the increase of Young's modulus on days 0–7 and slightly decreased on days 7–14 [28]. In addition, as cells are complex biological materials, their rheology is also an important biomechanics property. In recent years, optical tweezers, micropipette aspiration technique and AFM were widely used to investigate the rheology of suspension cells, such as red blood cells, neutrophils, etc. [43–45]. However, these studies mainly focus on the cell membrane tension and the adhesion between the cell membrane and the cytoskeleton, the understanding of cell fluidity at whole cell level and during stem cell differentiation is still lacking. In this study, we characterize the viscoelastic performance of hMSCs lineages by using two parameters, fluidity and creep compliance, which provides an important reference for quantifying the viscoelasticity of differentiated hMSCs.

Although considerable efforts have been made to investigate the biomechanical changes during stem cell differentiation, the correlation between the mechanical properties of the cytoskeleton and differentiation is still poorly understood. Some researchers have successfully intervened in cell differentiation by modulating the mechanical properties of the cytoskeleton. For example, Sen *et al*. claimed that continuous actin disruption with 0.1 µg ml^−1^ Cyto D would lead to the osteogenic lineage of hMSCs, and injection of Cyto D into the marrow space of live mice resulted in abundant bone formation within 1 week [46]. Mcbeath *et al*. indicated that disrupted actin with Cyto D increased adipogenesis and reduced osteogenesis of hMSCs, which emphasized the important role of the actin in the commitment process [47]. Lim *et al.* demonstrated that disruption of actin cytoskeleton induced chondrogenesis of mesenchymal cells by activating protein kinase C-α and inhibiting extracellular signal-regulated protein kinase signalling [48]. In addition to mesenchymal stem cells, disruption of the cytoskeleton also enhanced the adipogenic differentiation of embryonic stem cells [31]. Studies have focused on disrupting the cytoskeleton in the early stage (less than one week) of stem cell differentiation, ultimately enhancing the differentiation of particular lineages. In this study, the creep compliance and the fluidity of the differentiated hMSCs gradually increased in the early stage, which indicated that cytoskeletal mechanical properties were not just side effects but might be drivers of differentiated commitment.

**Figure 6.**
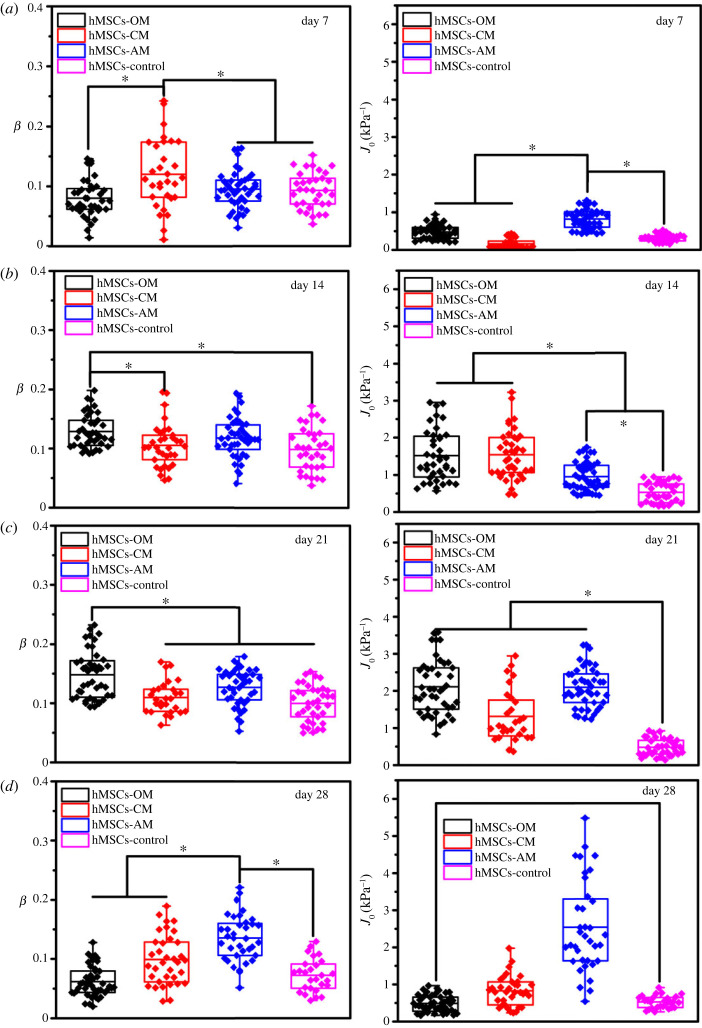
Long-term culture of the hMSCs from days 0 to 28 under the maintenance medium. (*a*) Fluidity degree and (*b*) creep compliance of the long-term cultured hMSCs from days 0 to 28. * (*p* < 0.05).

Significant works have recently been performed to investigate changes in the mechanical properties of long-term cultured cells. Some authors have suggested that long-term incubation and ageing of cells would cause the loss of mechanical stiffness [49]. However, the results are inconclusive, numerous studies based on linear elasticity theory have demonstrated that long-term culture without differentiation would not have a significant effect on the elasticity of hMSCs [6,49]. In this study, we found that the creep compliance of hMSCs increased from day 0 to 14 during the long-term incubation (*p* < 0.05). Accordingly, cell fluidity only increased before day 7 (table 4 and figure 6*a,b*). This phenomenon indicated that the composition and arrangement of the actin cytoskeleton would change in the early stage of long-term culture. The long-term culture of mesenchymal stem cells is accompanied by the shortening of telomere DNA and the dysregulation of mitochondrial function, which are thought to cause the accumulation of reactive oxygen species, and ultimately lead to damage to the cytoskeleton structure and affect the mechanical properties of cells [49].

**Table 4.** Least-square fitting parameters for the power-law rheology model of long-term cultured hMSCs.

			*J*_0_ (kPa^−1^)	*β*
		day 0	0.203 ± 0.122	0.607 ± 0.026
		day 7	0.316 ± 0.096	0.093 ± 0.030
hMSCs-control		day 14	0.528 ± 0.272	0.099 ± 0.037
		day 21	0.483 ± 0.222	0.100 ± 0.030
		day 28	0.529 ± 0.168	0.073 ± 0.028

It is worth mentioning that the results from the viscoelastic measurement of differentiated hMSCs showed a large standard deviation in the late stage of differentiation. This phenomenon was considered to be primarily caused by the presence of distinct cell subsets during differentiation. During osteodifferentiation, hMSCs contained several subpopulations, such as mesenchymal progenitors, preosteoblasts, osteoblasts and osteocytes. Previous studies have also shown that middle/deep-zone cells of chondrocytes are softer than superficial zone cells and comprise a greater percentage of the total cell population [50]. Despite the short duration of exposure time (1 h), performing the measurements at room temperature would also cause minor errors. These findings demonstrated that subpopulations of cells with different biomechanical properties existed in the test groups, which led to a large standard deviation. However, the measurement error of the outcome only increased the standard deviation, which was not expected to have a significant impact on the findings.

## 4. Conclusion

The viscoelastic behaviour of hMSCs differentiating into multiple lineages, including chondroblasts, osteoblasts and adipocytes, was determined. The PLR model was used to quantify the viscoelastic parameters at various days of cell differentiation. Osteodifferentiation and chondrogenic differentiation caused an increase in the creep compliance and the fluidity of the hMSCs at an early stage and a reduction of these parameters at the late stage. The reversal of the viscoelastic parameters during chondrogenic differentiation was earlier than during osteogenic differentiation. Adipogenic differentiation caused an increase in the creep compliance and the fluidity of the hMSCs. This study not only recharacterized the differentiation of hMSCs in terms of the creep compliance and the fluidity degree, but also provided a better understanding of the intracellular biophysical characteristics during the differentiation of hMSCs.

## Data Availability

The datasets and modelling code supporting the results of this article are provided in electronic supplementary material [51].
